# Lipophilic Extracts of *Portulaca oleracea* L.: Analysis of Bioactive Fatty Acids Targeting Microbial and Cancer Pathways

**DOI:** 10.3390/ph18040587

**Published:** 2025-04-17

**Authors:** Dejan Stojković, Jelena Živković, Stefani Bolevich, Gokhan Zengin, Mehmet Veysi Cetiz, Sergey Bolevich, Marina Soković

**Affiliations:** 1 Institute for Biological Research “Siniša Stanković”—National Institute of the Republic of Serbia, University of Belgrade, Bulevar despota Stefana 142, 11108 Belgrade, Serbia; 2 Institute for Medicinal Plants Research “Dr Josif Pancic”, Tadeusa Koscuska 1, 11000 Belgrade, Serbia; jelenazivkovic1@yahoo.com; 3 Department of Pathologic Physiology, First Moscow State Medical University I.M. Sechenov (Sechenov University), Trubetskaya Street, House 8, Building 2, 119991 Moscow, Russia; alistra555@mail.ru (S.B.); bolevich2011@yandex.ru (S.B.); 4 Department of Biology, Science Faculty, Selcuk University, Campus-Konya, 42250 Konya, Turkey; gokhanzengin@selcuk.edu.tr; 5 Department of Medical Biochemistry, Faculty of Medicine, Harran University, 63290 Sanliurfa, Turkey; mvcetiz@gmail.com

**Keywords:** purslane, fatty acids, cytotoxicity, antimicrobial, molecular informatics, polyunsaturated fatty acids

## Abstract

**Background/Objectives:** *Portulaca oleracea* L. (purslane) is a widely distributed plant known for its medicinal and nutritional properties. This study aims to evaluate the fatty acid composition and bioactivities of crude lipophilic extracts (chloroform/methanol 2:1) from purslane collected in Serbia and Greece, with a focus on its antimicrobial and anticancer potential. **Methods:** Chemical analysis was conducted to determine the fatty acid composition of the extracts. Antibacterial activity was assessed using standard microdilution assays, while antibiofilm assays evaluated the extracts’ ability to inhibit biofilm formation. Cytotoxicity was tested on cancer cell lines (MCF7, HeLa, CaCo2, HepG2) and normal keratinocyte cells (HaCaT). Molecular docking and dynamics simulations were performed to explore the interactions of bioactive fatty acids with microbial and cancer-related proteins. **Results:** The analysis revealed significant levels of polyunsaturated fatty acids, with linoleic acid as the predominant fatty acid in both samples (31.42% and 34.51%). The Greek extract exhibited stronger antibacterial activity than the Serbian extract, particularly against *Aspergillus versicolor*, *Pseudomonas aeruginosa*, and *Staphylococcus aureus*. Antibiofilm assays showed up to 89.54% destruction at MIC levels, with notable reductions in exopolysaccharide and extracellular DNA production, especially for Greek samples. Cytotoxicity testing indicated moderate effects on cancer cell lines (IC_50_ = 178.17–397.31 µg/mL) while being non-toxic to keratinocytes. Molecular docking identified strong interactions between key fatty acids and microbial and cancer-related proteins. **Conclusions:** These results highlight purslane’s potential as a source of bioactive compounds, particularly in antimicrobial and anticancer applications. The findings suggest that purslane extracts could be developed for therapeutic purposes targeting microbial infections and cancer.

## 1. Introduction

Common purslane (*Portulaca oleracea* L.) is a species of the Portulacaceae family and one of the most widely used medicinal and edible plants around the world [1]. In Mediterranean countries like Italy, Greece, and Spain, it is added to salads, soups, and stews, while in Morocco, it is collected as an edible wild plant. In India, purslane is used in curries and dal, and in Latin America, particularly Mexico, it is known as “verdolagas” and included in stews and tacos. Australian indigenous communities have traditionally consumed it, and in North America, it is gaining popularity in health-conscious diets, often used in gourmet dishes, smoothies, and salads [2]. It is considered a very invasive species and used to be ranked as the eight most common weed throughout the world [3]. Because it has always been a part of urban flora from ancient times, it is also referred to as a synanthropic species. Given its great adaptability to a wide range of climates and harsh conditions, purslane is a cosmopolitan weed that is found in many different parts of the world [4]. Although its origin is debatable, portulaca is said to be native in many parts of Europe, the far East, and South America [5].

**Figure 1:**
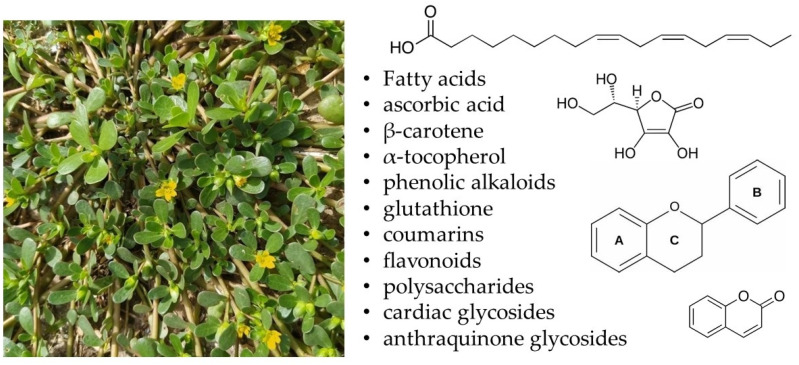
Main chemical compounds found in *Portulaca oleracea*.

Purslane is a rich source of α-linolenic acid, a vital omega-3 fatty acid, along with notable amounts of palmitic, oleic, and stearic acids, ascorbic acid, β-carotene, α-tocopherols, phenolic alkaloids, glutathione, coumarins, flavonoids, polysaccharides, cardiac glycosides, and anthraquinone glycosides (Figure 1) as well as many other beneficial compounds [6]. It provides substantial nutritional advantages because of its high content of omega-3 fatty acids and antioxidant qualities [7]. Additionally, studies have demonstrated its anti-inflammatory, antibacterial, and antidiabetic effects [8]. Type 2 diabetic mice have been treated with the polysaccharide from *P. oleracea*, which significantly lowered the mice’s fasting blood glucose, total cholesterol, and triglyceride levels [9,10].

Throughout history, medicinal plants have been utilized for a number of therapeutic purposes, including the management of microbial diseases [11]. Due to inappropriate use, such as using the wrong dosages and treatment periods, many bacterial strains are becoming more resistant to commercial antibiotics nowadays [12]. Antibiotics’ clinical efficacy is continuously decreasing due to the emergence of multidrug-resistant bacteria, which are a serious risk to human health [11]. Furthermore, using chemical antibacterial medications for therapy is costly and frequently linked to a host of negative side effects [13]. These issues highlight the need for innovations in antibiotic use, including better monitoring, prevention, diagnosis, and a reduction in drug misuse. In this context, medicinal plants offer a promising alternative as antibacterial agents against pathogenic, multidrug-resistant bacteria.

At the same time, finding an effective cancer treatment is becoming a more pressing issue. Long-term use of anticancer medications may cause cancer cells to become resistant [14]. The likelihood of multidrug resistance emerging in cancer patients, coupled with the severe side effects of chemotherapy, underscores the urgent need for new anticancer treatments. Substances derived from plants are promising candidates, as they may offer therapeutic benefits with minimal or no side effects.

The objective of this study was to conduct a comparative analysis of the fatty acid composition in plants from Serbia and Greece, while evaluating their biological activities, specifically antimicrobial and cytotoxic properties. Building upon these results, we performed molecular docking studies to gain deeper insights into the interaction mechanisms of the most abundant fatty acids identified in the extracts through phytochemical analysis. These molecular studies are crucial for understanding how these bioactive compounds interact at the molecular level, laying the foundations for the development of targeted therapeutic strategies based on the plant extracts’ fatty acid profiles.

## 2. Results and Discussion

### 2.1. Fatty Acid Composition

Purslane is considered a rich source of fatty acids, especially of omega-3 fatty acids such as α-linolenic acid, which is the richest plant source reported so far [15]. Therefore, its fatty acid content and composition could be considered a key quality factor for evaluation. The fatty acid composition of crude lipophilic (chloroform/methanol 2:1) extracts is presented in Table 1 for purslane samples collected in Greece and Serbia. Common names of fatty acids found in the sample are presented in the Supplementary File Table S1.

From the fatty acids, the most dominant was linoleic acid (C18:2n6) in both samples (31.42% and 34.51% of total fatty acids, respectively), followed by α-linolenic acid (C18:3n3) and palmitic acid (C16:0). Oliveira et al. [16] have reported the same fatty acids to be the most abundant. However, they detected significantly lower percentages for linoleic acid (4.00–6.31%). This difference may be attributed to the fact that they analyzed the leaves separately from the stems, whereas in this study the results refer to whole plants (stems and leaves) since they are both edible.

Polyunsaturated fatty acids (PUFAs) accounted for the highest fraction of total fatty acids (FAs) (54.12 and 51.32%). Palmitic acid was detected at significant amounts (20.32–21.87%). Moreover, linoleic acid was the prevailing fatty acid for both samples (31.42–34.51%) in the samples from Serbia and Greece, respectively.

It can be concluded that both of the samples were rich source of polyunsaturated fatty acids. These results indicate that the tested samples had some differences concerning chemical composition. Omega-3 fatty acid (α-linolenic acid) content is considered the main quality feature of purslane. Namely, the omega-6 to omega-3 ratio is crucial for human health, ideally ranging from 1:1 to 10:1, with ratios of 1:1, 2:1, and 4:1 particularly recommended for medicinal purposes [17]. In our samples, this ratio was almost 1:1. Both of the samples, from Greece and Serbia, might be suggested for cultivation, since they have important means for food products of high quality and increased added value, in terms of high omega-3 fatty acid content.

### 2.2. Antimicrobial Activity

The antimicrobial effectiveness of *P. oleracea* crude lipophilic (chloroform/methanol 2:1) extracts collected from Serbia and Greece against various bacterial and fungal strains is presented in Table 2. The results are categorized into minimum inhibitory concentrations (MICs) and minimum bactericidal/fungicidal concentrations (MBCs/MFCs).

For the tested bacterial strains, which include *Staphylococcus aureus*, *Bacillus cereus*, *Listeria monocytogenes*, *Micrococcus flavus*, *Pseudomonas aeruginosa*, *Escherichia coli*, *Salmonella typhimurium*, and *Enterobacter cloacae*, the extracts from Greece exhibit moderate antibacterial activity. The MIC values for the Greek samples range from 0.59 to 4.74 μg/mL, with corresponding MBC values generally double the MIC, suggesting a moderate level of effectiveness. The MIC for *Staphylococcus aureus* is recorded at 0.59 μg/mL, while the MBC reaches 1.18 μg/mL. In comparison, the Serbian extracts show slightly higher MIC and MBC values, particularly for *Listeria monocytogenes* and *Salmonella typhimurium*, indicating lower antibacterial potency. Standard antibiotics such as streptomycin and ampicillin show significantly lower MIC and MBC values, highlighting their greater effectiveness against these bacterial strains compared to the purslane extracts. The obtained data are in accordance with previous results. Namely, El-Hack et al. [7] demonstrated that purslane ethanolic extract was more potent against Gram-positive bacteria than Gram-negative bacteria. Also, they showed that the mentioned extract was more effective as an antibacterial than an antifungal. Othman (2017) [18] pointed out significant activity of purslane seed fatty oil against *S. epidermidis* and *E. coli* and connected this activity with the high content of omega-3 fatty acids. The extract obtained using carbon dioxide extraction of *Portulaca oleracea* has demonstrated effectiveness against important microorganisms, including *Escherichia coli* and *Staphylococcus aureus* [19]. In our study, the analysis of fungal strains, including *Aspergillus fumigatus*, *Aspergillus versicolor*, *Aspergillus ochraceus*, *Aspergillus niger*, *Trichoderma viride*, *Penicillium funiculosum*, *Penicillium ochrochloron*, and *Penicillium verrucosum* var. *cyclopium*, reveals a similar trend. The Greek samples demonstrate broader antifungal activity, with MIC values ranging from 0.025 to 2.38 μg/mL, showing the lowest MIC against *Aspergillus versicolor* at 0.025 μg/mL. The MFC values for these samples tend to be higher, indicating that more potent concentrations are required to achieve fungicidal effects. Conversely, the Serbian samples yield higher MIC values, suggesting lower antifungal efficacy. When compared to standard antifungal agents such as ketoconazole and bifonazole, which exhibit lower MIC and MFC values, it becomes evident that these conventional drugs are more effective than purslane extracts in combating fungal infections. The antifungal potential of *P. oleracea* was also investigated by other authors. In a study conducted by Bauerjee and Mukherjee [20], chloroform extracts of *P. oleracea* demonstrated strong activity against *Rhizopus artocarpi*, a fungal species known to cause jackfruit rotting. While the *P. oleracea* extracts from Greece display slightly better antimicrobial activity than those from Serbia, both exhibit moderate efficacy against certain bacterial strains and fungi. However, their effectiveness is still considerably lower than that of standard antibiotics and antifungals, suggesting that while purslane may have some antimicrobial potential, it is not as potent as established therapeutic agents. Several studies have explored the antimicrobial mechanism of fatty acids in *S. aureus*. Zheng et al. [21] pointed out that linoleic acid inhibited bacterial enoyl–acyl carrier protein reductase (FabI), a crucial enzyme in bacterial fatty acid synthesis, making it a promising target for antibacterial drugs. According to Kelsey et al. (2006) [22], the seven most potent antimicrobial fatty acids include lauric acid (C12:0), capric acid (C10:0), myristic acid (C14:0), and polyunsaturated linoleic acid (C18:2). Similarly, when used in combination with the antibiotic compound erythromycin, both linoleic acid and oleic acid from *P. oleracea* demonstrated a synergistic effect in inhibiting the growth of methicillin-resistant *S. aureus* [23]. Linoleic acid at 16 μg/mL and oleic acid at 32 μg/mL reduced the MIC of erythromycin from 256 to 16 μg/mL (an 8-fold decrease) and from 256 to 32 μg/mL (a 4-fold decrease), respectively. Nguyen et al. [24] highlighted that although *S. aureus* cannot produce unsaturated fatty acids on its own, it incorporates long-chain unsaturated fatty acids into its lipoproteins, which helps the bacteria be more easily recognized by the body’s immune system [24].

### 2.3. Antibiofilm Activity

*S. aureus* is normally found as a harmless part of the human microbiota, living on the skin, mucous membranes, and in the reproductive tract [25]. However, this bacterium can sometimes cause a range of infections, from mild to severe, including life-threatening diseases. Strains that are resistant to antibiotics, such as methicillin-resistant *S. aureus* (MRSA), pose a significant challenge in healthcare settings. One key strategy that *S. aureus* uses to grow and survive in host tissues is forming biofilms. In this state, the bacteria are embedded in a self-produced extracellular matrix, which is mainly made up of polysaccharides, proteins, and nucleic acids [26]. Previous studies on the antibiofilm activity of various purslane extracts highlighted their potential as an alternative antibiofilm treatment. Ma et al. [27] exhibited that *P. oleracea* aqueous extract reduced the bacterial density on a biofilm’s surface by causing some bacteria to die off or causing their transition to a planktonic state. As a result, the immune system was able to target and eliminate the surviving cells within the biofilm. The antibiofilm activity of purslane crude lipophilic (chloroform/methanol 2:1) extracts is presented in Table 3. In our study, the crystal violet assay revealed a strong potential of both tested purslane extracts to inhibit biofilm formation. While the highest inhibition rates (88% and 90% for extracts from Greece and Serbia, respectively) were observed at the MIC, it is important to note that significant and statistically reliable biofilm inhibition was also detected at ½ MIC (75% and 81% for the extracts from Greece and Serbia, respectively) and even at ¼ MIC. Yuyama et al. [28] demonstrated that most fatty acids could inhibit biofilm formation in Gram-positive bacteria at sub-MIC concentrations. In the same study, with the exception of palmitic acid and oleic acid, all the tested fatty acids were able to reduce biofilm formation in *S. aureus*, with some even lowering it to a concentration of 4 µg mL^−1^. Kim et al. [29] showed that polyunsaturated fatty acid, linolenic acid, inhibited the formation of *Pseudomonas aeruginosa* biofilm without affecting bacterial growth. Antimicrobial studies of natural products often do not reveal the specific mechanisms through which these compounds exert their effects. However, to fully harness the biological potential of a product, it is essential to understand the mechanisms it uses.

Biofilms are defined as clusters of microorganisms or multicellular communities that are encased in an extracellular matrix produced by the microorganisms themselves [30]. The matrix surrounding the microorganisms is made up of extracellular polymeric substances, which usually include sugars, proteins, lipids, and DNA. An antibiofilm strategy primarily aims to target quorum sensing, degrade the extracellular matrix, prevent microbial adherence, and eliminate persister cells, all of which are key components of an effective antibiofilm approach. The Congo red binding assay is used to assess the production of exopolysaccharides (EPSs) in biofilms, and in our study, it showed a significant decrease in exopolysaccharide production in the biofilm matrix. Exopolysaccharides are important for the biofilm’s protective barrier. When purslane extracts (½ MIC) were applied in our study (Table 4), EPS production dropped by 56% and 42% for the extracts from Greece and Serbia, respectively. This suggests that reducing exopolysaccharide production may be one way these extracts work to disrupt biofilms. This reduction indicates that the extracts might interfere with their production or export, weakening the biofilm and making the bacterial cells more vulnerable. Along with extracellular proteins and exopolysaccharides, extracellular DNA (eDNA) is a prevalent component of the extracellular matrix in biofilms produced by various bacteria [31]. At the early stages, eDNA disperses extensively across the substrate surface, facilitating coordinated movement through the channel network by maintaining precise cell alignments. Disrupting any phase of biofilm’s multicellular structure formation, including eDNA production inhibition, could enhance the effectiveness of therapeutics and host defenses, potentially leading to improved treatment outcomes. Eliminating eDNA weakens the biofilm matrix, and although this may not fully disperse the biofilm, it can make it more vulnerable to antibiotics [32]. eDNA plays a crucial role in Gram-positive *Staphylococcus* biofilms. Nearly all strains of *S. aureus* contain eDNA in their biofilms, with its concentration varying depending on the culture conditions [33]. Both tested extracts in the current study significantly decreased eDNA production, with a higher reduction recorded for the sample from Greece (76%) (Table 5). This result suggests that it could be the main mechanism behind the activity. Previous studies showed that fatty acids could provide a promising alternative to conventional antibiotics in the fight against various bacterial infections. Wei et al. [34] showed that γ-linolenic acid has the potential to significantly downregulate the expression of the *atlA* gene in vancomycin-resistant *Enterococcus faecium* strains, which subsequently inhibited eDNA release and led to the eradication of biofilms. Previous research showed that fatty acids, such as linoleic acid, are known to disrupt bacterial membrane integrity, resulting in increased membrane permeability and leakage of cellular contents. This disruption can enhance bacterial susceptibility to antimicrobial agents and destabilize biofilms [35]. At the same time, linoleic acid has been shown to affect quorum sensing in bacteria, a process that controls biofilm formation and eDNA production. By disrupting quorum sensing pathways, linoleic acid may reduce eDNA production [36].

### 2.4. Cytotoxicity

The cytotoxicity data presented in Table 6 provides insights into the effects of *P. oleracea* crude lipophilic (chloroform/methanol 2:1) extracts from Greece and Serbia on various cell lines, measured by the half-maximal inhibitory concentration (IC_50_) values. For the HaCaT cell line, which represents human keratinocytes, both Greek and Serbian extracts show an IC_50_ value greater than 400 µg/mL. This suggests that purslane extracts are non-cytotoxic to HaCaT cells at these concentrations, indicating a favorable safety profile for this particular cell line. In the MCF7 cell line, used as a model for breast cancer research, the IC_50_ values for the Greek and Serbian extracts were 397.31 µg/mL and 374.33 µg/mL, respectively. This indicates that both extracts exhibit moderate cytotoxicity towards MCF7 cells, with the Serbian extract showing slightly greater potency. For the HeLa cell line, representing cervical cancer, similar to MCF7, both extracts demonstrate moderate cytotoxicity, with a slight advantage for the Greek extract. The CaCo2 cell line, which models human colorectal cancer, exhibits lower IC_50_ values. Both extracts show higher cytotoxicity compared to the previous cell lines, with the Serbian extract being marginally more potent. Lastly, the IC_50_ values are 292.37 µg/mL for the Greek extract and 272.36 µg/mL for the Serbian extract. For the HepG2 cell line, which represents human liver cancer, both extracts display moderate cytotoxicity, with the Serbian extract showing a slightly stronger effect. While the extracts from both Greece and Serbia show varying degrees of cytotoxicity across different cell lines, they remain largely non-toxic to normal cells (HaCaT) and exhibit moderate cytotoxic effects on cancer cell lines (MCF7, HeLa, CaCo2, and HepG2). The Serbian extracts tend to show slightly higher potency compared to the Greek extracts, particularly in the CaCo2 and HepG2 cell lines, suggesting potential for further investigation into their therapeutic applications. Previous in vitro studies have highlighted the beneficial effects of purslane seeds and seed oil on the HeLa and A549 cell lines. Al-Sheddi et al. [37] found that purslane seed oil may have notable cytotoxic effects against human liver (HepG2) and lung cancer (A-549) cell lines. Additionally, Asif [38] proposed that the anticancer properties of seed oils could be linked to polyunsaturated fatty acids, particularly a group of conjugated isomers of linoleic acid. Petropoulos et al. [39] demonstrated the absence of hepatotoxicity in cytotoxic assays, with GI_50_ values exceeding 400 μg/mL across all harvesting stages and parts of the purslane plant. Unsaturated fatty acids (UFAs), such as linoleic and linolenic acids, two dominant fatty acids in the tested purslane samples, have been shown to exhibit cytotoxic effects or growth inhibition against various malignant cell lines [40].

Over the past two decades, these effects have been extensively studied, with evidence suggesting a selective action on malignant cells compared to normal cells. Banfi et al. [41] demonstrated that the incorporation of linoleic acid into HepG2 cells enhances the secretion of plasminogen activator inhibitor type 1 into the surrounding medium, leading to a reduction in the fibrinolytic potential of the cells.

### 2.5. Bacterial Invasion

**Figure 2:**
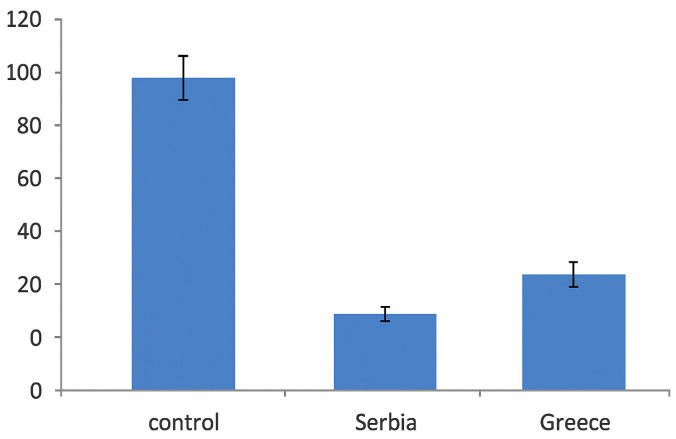
Effects of purslane extracts on the invasion capacity of *S. aureus* in HaCaT cells. The results are expressed as average values of three replicates ± SD.

While *S. aureus* is primarily recognized as an extracellular pathogen, it has been demonstrated to invade and persist within endothelial cells. The invasion assay was used to assess the ability of *S. aureus* to penetrate keratinocytes in tissue culture. This assay is founded on the principle that intracellular bacteria are shielded from the antimicrobial action of the investigated sample, which is added to the medium to eliminate extracellular or adherent bacteria [42]. According to our results, purslane samples showed medium invasion inhibition, which was slightly higher for the sample from Greece (around 30%). The results are presented in Figure 2 as the percentage of bacterial invasion of *S. aureus* relative to the bacterial control group (100% invasion), which was not treated with *P. oleracea* extracts. Several studies have shown that certain fatty acids, particularly unsaturated fatty acids like linoleic acid, can interfere with the ability of *S. aureus* to invade host cells, including keratinocytes [43]. This inhibition probably occurs through multiple mechanisms, including alterations in bacterial membrane fluidity, the disruption of virulence factors, and interference with cell signaling pathways involved in bacterial invasion.

### 2.6. Evaluating Docking Outcomes: Ligand Binding Energies and Interaction Profiles

**Figure 3:**
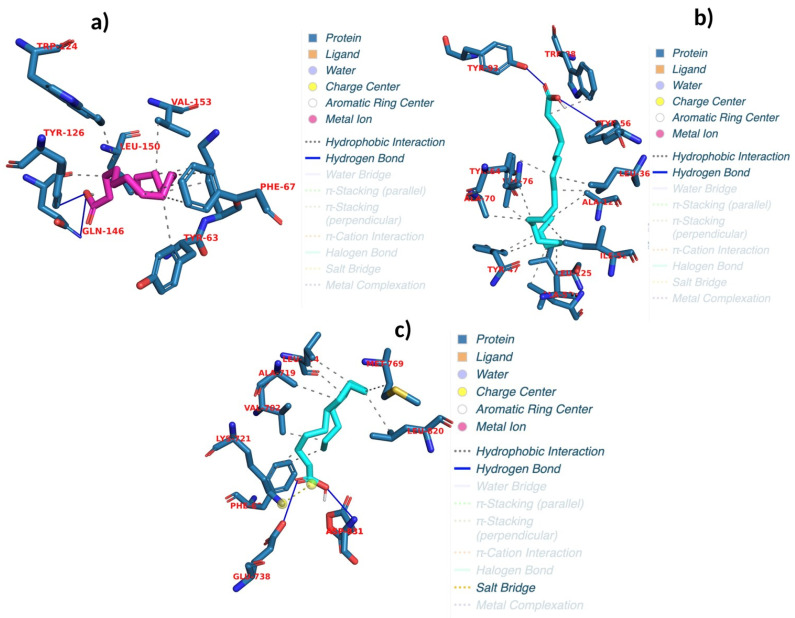
Binding interactions of proteins with compounds showing the best binding energy. (**a**) Interaction between palmitic acid and PrfA. (**b**) Interaction between oleic acid and LasR. (**c**) Interaction between palmitoleic acid and EGFR.

This study employed a comprehensive approach to assess the antimicrobial and anticancer properties of bioactive compounds identified in purslane, targeting a diverse range of microbial and cancer-related proteins. Chemical profiling revealed the presence of several notable compounds, including linolenic acid, palmitic acid, oleic acid, palmitoleic acid, and linoleic acid. This study identified key bacterial proteins, including those derived from *Listeria monocytogenes* (LPI-PLC, InlA, PrfA), *Bacillus cereus* (Bc II, PatB1, PadR, TubR), *Pseudomonas aeruginosa* (FabZ, LasR, LpxC, beta-lactamase Oxa-10), *Staphylococcus aureus* (30S ribosome S3, dihydropteroate synthase, gyrase B, MurE, transpeptidase), and *Escherichia coli* (30S ribosome S3; additionally, dihydropteroate synthase, gyrase B, MurE, and transpeptidase). Proteins associated with colon adenocarcinoma (c-FOS, CDK4, CDKN1A, CyclinD1, IFN-γ, IL-2, and IRS-1) were also examined. The interactions between these compounds and NF-κB p65, TGF-β1, and E2F1, as well as between the compounds and lung adenocarcinoma-related proteins such as AKT-1, CDK2, cIAP1BIR3, EGFR, MDM2, PI3K delta, and Stat3, were then analyzed. The objective of the investigation was to elucidate the potential antibacterial and anticancer activities of the compounds by targeting bacterial virulence, cell wall biosynthesis, antibiotic resistance, and cancer progression pathways. The docking analysis revealed binding energies ranging from −8 to −4.4 kcal/mol, with RMSD values between 1.09 and 41.9 (Table 1, Table S2 and Table S3). Linolenic acid exhibited significant binding to AKT-1, forming two hydrogen bonds at PHE A:293 and GLY A:294, with a binding energy of −5.3 kcal/mol and an RMSD of 1.7, indicating its potential as an AKT-1 inhibitor. Palmitoleic acid showed favorable binding affinity to EGFR, forming four hydrogen bonds at GLU A:738 and ASP A:831 (with repeated interactions at ASP A:831), achieving a binding energy of −5.2 kcal/mol and an RMSD of 1.9. Oleic acid demonstrated strong inhibitory activity against LasR, forming two hydrogen bonds at TYR E:56 and TYR E:93, with a binding energy of −7.7 kcal/mol and an RMSD of 1.6 (Figure 3b). Palmitoleic acid demonstrated strong inhibitory activity against EGFR, forming four hydrogen bonds at GLU A:738 and ASP A:831 (3), with a binding energy of −5 kcal/mol and an RMSD of 1.9 (Figure 3c). Furthermore, palmitic acid effectively interacted with PrfA, forming two hydrogen bonds at TYR A:126 and GLN A:146, with a binding energy of −6.5 kcal/mol and an RMSD of 1.7, highlighting its potential inhibitory effects (Figure 3a and Table 7). These findings provide valuable insights into the bioactivity of these compounds and their prospective roles in antimicrobial and anticancer therapies.

### 2.7. Binding Free Energy Analysis: MM/PBSA Results and Implications for Ligand Efficacy

In this study, the effect of energy components on binding stability was evaluated by calculating and analyzing a series of protein–ligand complexes. The investigation focused on several important energy parameters, including van der Waals interaction (VDWAALS), electrostatic energy (EEL), polar solvation energy (EGB), surface tension (ESURF), gas phase energy (GGAS), solvation energy (GSOLV), and total energy (TOTAL). The evaluation of cancer-related enzyme activities of compound derived from purslane was performed using MM/PBSA binding free energy calculations in conjunction with molecular dynamics simulations. Four complexes were selected for further analysis based on factors such as low root mean square deviation (RMSD), high binding energy, and the number of hydrogen bonds formed. The complexes selected for further analysis are palmitic acid_PrfA, oleic acid_LasR, palmitoleic acid_EGFR, and linolenic acid_AKT-1, as shown in Table S4. The complexes chosen for further analysis, based on their energy profiles and stability, are described below: Palmitic acid_PrfA had an overall energy value of −25.19 kcal/mol, with a good contribution from van der Waals contacts (VDWAALS = −35.08 kcal/mol) and a positive binding profile with an additional boost from electrostatic interactions (EEL = −9.59 kcal/mol). Its solvation energy (GSOLV = 19.48 kcal/mol) opposed the interaction, indicating a stable binding conformation. Oleic acid_LasR complexed with a slightly higher total energy value of −22.36 kcal/mol, where strong van der Waals interaction (VDWAALS = −36.24 kcal/mol) and strong electrostatic contributions (EEL = 64.24 kcal/mol) were observed. However, the solvation energy (GSOLV = −50.36 kcal/mol) and overall interaction profile suggest stable but dynamic binding behavior. In the palmitoleic acid_EGFR complex, the overall energy value was registered at −17.58 kcal/mol, which is marked by relatively balanced van der Waals contacts (VDWAALS = −34.94 kcal/mol) and electrostatic contacts (EEL = 33.52 kcal/mol). However, the solvation energy (GSOLV = −16.16 kcal/mol) rendered a less favorable net energetic profile. The lowest binding energy was recorded for linolenic acid_AKT-1 with a value of −18.71 kcal/mol, characterized by moderate van der Waals forces (VDWAALS = −23.54 kcal/mol) as well as considerable electrostatic effects (EEL = −36.07 kcal/mol) and solvation energy (GSOLV = 40.89 kcal/mol), indicating interaction that is stable but potent to a lesser extent (Table S4). The results point out the importance of van der Waals forces coupled with the balance of electrostatic and solvation energy contributions in determining the stability and binding potential of the complexes above. Of these, palmitic acid_PrfA and oleic acid_LasR stand out as being particularly stable, while linolenic acid_AKT-1 and palmitoleic acid_EGFR exhibit highly dynamic interaction profiles, which would require further exploration using molecular dynamics. Its findings have shown that these compounds have promising therapeutic capabilities to target certain proteins and pathways.

### 2.8. Stability and Flexibility in Molecular Dynamics Simulation

**Figure 4:**
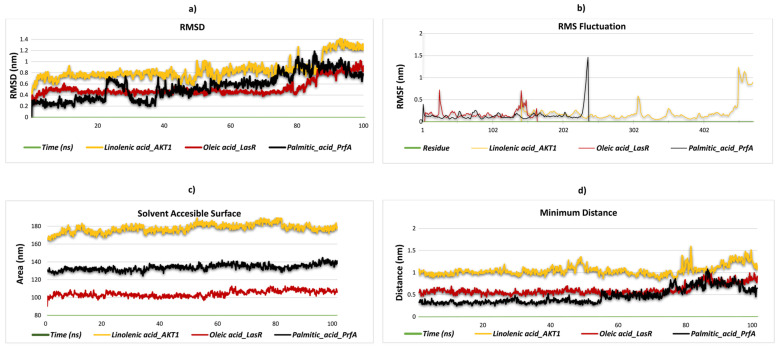
Presentation of molecular dynamics simulations in graphical form: (**a**) RMSD of palmitic acid_PrfA, oleic acid_LasR, and linolenic acid_AKT-1 complexes. (**b**) RMSF palmitic acid_PrfA, oleic acid_LasR, and linolenic acid_AKT-1 complexes. (**c**) Solvent accessibility of palmitic acid_PrfA, oleic acid_LasR, and linolenic acid_AKT-1 complexes. (**d**) Minimum distance of palmitic acid_PrfA, oleic acid_LasR, and linolenic acid_AKT-1 complexes.

**Figure 5:**
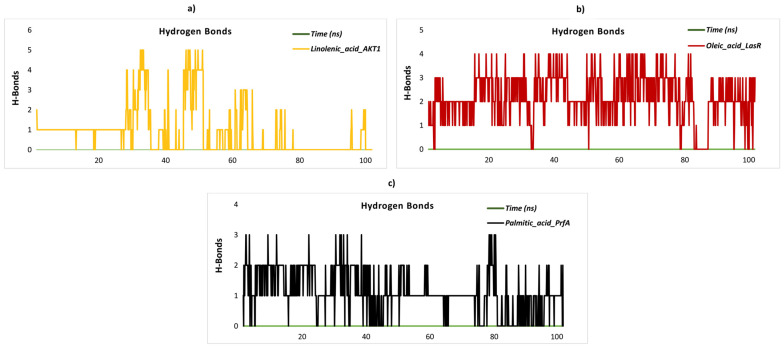
Hydrogen bonds in complexes. (**a**) Hydrogen bonds in linolenic acid_AKT-1 complex. (**b**) Hydrogen bonds in oleic_acid_LasR complex. (**c**) Hydrogen bonds in palmitic acid_PrfA complex.

This study aims to uncover potential therapeutic drugs through a thorough investigation of the molecular interactions between specific ligands and target proteins, with an emphasis on clarifying their binding sites. Based on important criteria such as the presence of hydrogen-bonding residues, the results of MM/PBSA binding free energy calculations, and molecular docking scores, four complexes were selected for evaluation. Palmitic acid_PrfA, oleic acid_LasR, and linolenic acid_AKT-1, four ligand–protein complexes, showed robust selectivity and stability in their interactions. These complexes were subjected to molecular dynamics (MD) simulations, which provided greater insight into their biological efficacy and protein binding capacities to better evaluate their potential as therapeutic agents. The structural stability of three distinct ligand–protein complexes was evaluated through the analysis of their RMSD values, which were obtained from molecular dynamics simulations conducted over 100 ns. The linolenic acid_AKT1 complex exhibits notable structural variability, with RMSD values fluctuating between 0.4 and 1.3 nm throughout the simulation. This suggests that the complex is less stable in terms of its structural conformation. The palmitic acid_PrfA complex displays moderate stability, with RMSD values oscillating between 0.1 and 1.0 nm. While it is not relatively stable, it does not reach the level of structural integrity observed for the oleic acid_LasR complex. In contrast, the oleic acid_LasR complex exhibits a more stable structural conformation, with RMSD values remaining between 0.3 and 0.8 nm. The stable RMSD profile observed for this complex throughout the simulation period indicates that it maintains structural integrity in the binding region and may exhibit stronger interactions (Figure 4a). The RMSF plot serves to illustrate the flexibility of residues in protein complexes. The RMSF profile of the linolenic acid_AKT1 complex exhibits a maximum value of 0.5 nm in the latter stages of the simulation, indicating increased flexibility in these regions. This is likely to correspond to structurally disordered areas. Similarly, the oleic acid_LasR complex also displays notable flexibility between residues 25–50, with the RMSF values reaching up to 0.7 nm towards the conclusion of the simulation. This suggests that these regions are undergoing dynamic movements. In the palmitic acid_PrfA complex, while overall flexibility remains low, RMSF values reach 0.4 nm in the later stages of the simulation. As the binding site of the ligand is unknown, it is more probable that these increases are associated with flexibility in terminal or disordered regions of the protein rather than ligand binding. This suggests that these regions display increased dynamic mobility and potentially reduced stabilization. Further clarification of the significance of these increases may be obtained through additional structural analyses (Figure 4b). The SASA analysis of the ligand–protein complexes revealed significant differences in solvent exposure. The linolenic acid_AKT1 complex exhibits the highest average SASA values (~170 nm^2^), indicating the largest solvent exposure and a relatively open structure. In contrast, the oleic acid_LasR complex exhibits consistently lower SASA values, suggesting a more compact structure with reduced interaction with the solvent. The palmitic acid_PrfA complex exhibits intermediate SASA values, indicating a moderate degree of solvent exposure. These findings indicate that the linolenic acid_AKT1 complex interacts extensively with the solvent, whereas the oleic acid_LasR complex is more solvent-isolated, which may contribute to differences in structural dynamics and stability. The palmitic acid_PrfA complex represents a balance between these two extremes, exhibiting moderate stability and interaction with the solvent. A 100 ns simulation was employed for the SASA analysis of the ligand–protein complexes, with the objective of evaluating the dynamics of solvent exposure. The linolenic acid_AKT1 complex exhibited the highest mean SASA values (~170 nm^2^) throughout the simulation, with notable fluctuations observed over time. This suggests that the complex assumes a relatively open structure, with considerable solvent exposure and dynamic conformational alterations. In contrast, the oleic acid_LasR complex exhibited consistently lower SASA values with minimal fluctuations, indicating a more compact and stable structure with reduced solvent interaction. The palmitic acid_PrfA complex exhibited intermediate SASA values, indicating a moderate degree of solvent exposure with minimal fluctuations during the simulation. These findings indicate that the linolenic acid_AKT1 complex interacts extensively with the solvent, likely due to dynamic structural changes. In contrast, the oleic acid_LasR complex remains more solvent-isolated and structurally stable. The palmitic acid_PrfA complex, situated between these two extremes, demonstrates a balance of moderate stability and solvent interaction (Figure 4c). The minimum distance between atoms in the binding region provides insights into the structural stability of ligand–protein complexes. The linolenic acid_AKT1 complex displays a notable range of fluctuations in the minimum distance between 0.9 and 1.4 nm throughout the simulation, indicating considerable mobility in the binding region. Nevertheless, this mobility becomes more pronounced after 80 ns, suggesting an increase in flexibility in the latter stages of the simulation. This suggests that the complex exhibits considerable flexibility in its binding conformation over time. In contrast, the oleic acid_LasR complex exhibits a more stable profile, with minimum distances predominantly fluctuating between 0.4 and 0.7 nm, indicating a compact and stable binding conformation. Notwithstanding minor fluctuations, the overall stability of this complex is noteworthy. The palmitic acid_PrfA complex displays the most stable behavior, with minimum distances consistently ranging between 0.2 and 0.5 nm, indicating a highly compact and stable binding conformation. This suggests that the binding region in this complex remains tightly packed and undergoes minimal dynamic changes during the simulation. These findings indicate that the palmitic acid_PrfA complex exhibits the most compact and stable structure in its binding region, whereas the linolenic acid_AKT1 complex displays greater flexibility and dynamic behavior in its binding conformation, especially in the later stages of the simulation (Figure 4d). The minimum distance between atoms in the binding region offers crucial insights into the structural stability of ligand–protein complexes. The linolenic acid_AKT1 complex displays fluctuations in the minimum distance, with values ranging between 0.9 and 1.6 nm throughout the simulation. It is noteworthy that a considerable increase in the distance is observed after 80 ns, reaching up to 1.6 nm, which indicates increased flexibility and relaxation in the binding region during the latter stages of the simulation. The oleic acid_LasR complex typically exhibits minimum distances between 0.4 and 1.0 nm, indicating a moderate degree of stability in the binding region. Nevertheless, a gradual increase in the distances is observed after 80 ns, indicating a slight degree of flexibility in the binding region. The palmitic acid_PrfA complex exhibits the most compact binding region, with minimum distances consistently ranging between 0.2 and 0.5 nm for the majority of the simulation. Following an initial increase in distance after 80 ns, the complex undergoes a decrease in distance after 90 ns, thereby restoring the compact nature of the binding region. In general, all complexes display increased flexibility after 80 ns. However, the palmitic acid_PrfA complex demonstrates the capacity to regain its compact structure, while the linolenic acid_AKT1 complex exhibits the most pronounced structural relaxation, indicating a high degree of flexibility. Hydrogen bond analysis offers invaluable insights into the structural stability and dynamic behavior of ligand–protein complexes. In the case of the linolenic acid_AKT1 complex, the number of hydrogen bonds exhibited considerable variation throughout the course of the simulation. The number of hydrogen bonds initially remained stable between one and two bonds, but it subsequently declined significantly, reaching zero after 80 ns. This indicates a loss of binding stability in the later stages (Figure 5a). Similarly, the oleic acid_LasR complex exhibited moderate fluctuations, with hydrogen bond numbers primarily ranging from one to three during the simulation. However, a precipitous decline to zero bonds was observed after 80 ns, underscoring a destabilization in the binding region (Figure 5b). In contrast, the palmitic acid_PrfA complex exhibited a relatively lower number of hydrogen bonds, predominantly between one and two throughout the simulation, with occasional decreases to zero bonds. This trend persisted after 80 ns, with the number of hydrogen bonds frequently falling to zero, indicating notable instability (Figure 5c). These observations collectively suggest that while all three complexes maintain moderate binding stability in the initial stages, significant losses in hydrogen bonding occur in the later simulation stages, particularly after 80 ns. This indicates reduced structural stability and potential conformational changes in the binding regions.

## 3. Materials and Methods

### 3.1. Chemicals and Reagents

The chemicals and reagents used in this study included chloroform, methanol, phosphate-buffered saline (PBS, Sigma-Aldrich, Darmstadt, Germany), crystal violet (Bio-Merieux, Craponne, France), 96% ethanol (Zorka, Bezdan, Serbia), dimethyl sulfoxide (DMSO, Sigma-Aldrich, Steinheim, Germany), Congo red solution (Sigma-Aldrich, Steinheim, Germany), Dulbecco’s Modified Eagle Medium (DMEM, Gibco, Hennigsdorf, Geremany), Fetal bovine serum (FBS, Gibco, Hennigsdorf, Geremany), 2 mM L-glutamine (Invitrogen, Darmstadt, Germany), 1% antibiotic-antimycotic (Invitrogen, Darmstadt, Germany), 1% Tween-20 (Sigma-Aldrich, Darmstadt, Germany), ellipticine (Sigma-Aldrich, Darmstadt, Germany), and Trypton Soy Broth (TSB, Torlak, Belgrade, Serbia). All chemicals were of reagent grade and used as received.

### 3.2. Collection of the Samples

*Purslane* was collected from two different localities, with one sample from central Serbia and the other sample originating from northern Greece. Plant material was collected during the flowering period (June, 2022) and identified by morphological characteristics by botanist dr. Dejan Stojković. The plant material was deposited in the local herbarium of the Institute for Biological Research ‘Siniša Stanković’ under the voucher numbers POCS1/2022 and PONG2/2022. For all the analyses, plant tissue samples (whole aerial parts) were taken from 15 plants from each genotype, and all the samples were pulled in one and stored at deep freezing conditions (−80 °C) and freeze-dried prior to analysis. The freeze-dried samples were powdered with a pestle and mortar and divided into three samples for further analysis.

### 3.3. Extraction of the Samples

The ground samples of purslane were sieved to 0.75 mm, placed in a thimble (4 cm × 17 cm), and subjected to Soxhlet extraction with a chloroform/methanol mixture (2:1) for 8 h. The extraction solvent was further evaporated in a rotary vacuum evaporator, and the extract was stored in a sealed container in the refrigerator until further use.

### 3.4. Fatty Acids

Following a transesterification process as previously mentioned [44], fatty acids were identified using a gas chromatographer (DANI 1000) fitted with a split/splitless injector and a flame ionization detector (GC-FID). By comparing the relative retention periods of fatty acid methyl ester (FAME) peaks from samples with standards, fatty acid identification was accomplished. The CSW 1.7 program (DataApex 1.7) was used to record and process the results. Each fatty acid’s relative percentage was used to express the results.

### 3.5. Antimicrobial Activity

To evaluate the antibacterial potential of the optimized extract, a previous methodology described by Soković et al. [45] was used, and three Gram-positive bacteria were tested, namely, *Staphylococcus aureus* (ATCC 11632), *Bacillus cereus* (clinical isolate), *Listeria monocytogenes* (NCTC 7973), as well as three following Gram-negative bacteria: *Escherichia coli* (ATCC 25922), *Salmonella enterica* serovar. Typhimurium (ATCC 13311) and *Enterobacter cloacae* (ATCC 35030) were used to determine potential antimicrobial activity of the samples. Additionally, to determine the antifungal activity, the methodology described by Soković and van Griensven [46] was used and applied to six micromycetes: *Aspergillus fumigatus* (human isolate), *Aspergillus niger* (ATCC 6275), *Aspergillus versicolor* (ATCC11730), *Penicillium funiculosum* (ATCC 36839), *Trichoderma viride* (IAM 5061), and *Penicillium verrucosum* var. *cyclopium* (food isolate). The findings, which were quantified in mg/mL, were shown as the minimum inhibitory concentration (MIC), minimum bactericidal concentration (MBC), and minimum fungicidal concentration (MFC). The commercial antibiotics streptomycin, ampicillin, ketoconazole, and bifonazole were employed as positive controls, while solvent served as a negative control.

### 3.6. Antibiofilm Activity

The impact of the extracts on *S. aureus* biofilms was determined as described previously in Smiljković et al., 2018 [47], with some modifications. In 96-well microtiter plates with an adhesive bottom (Sarstedt, Nümbrecht, Germany), *S. aureus* was cultured for 24 h at 37 °C in triptic soy broth containing 2% glucose in order to form biofilms. Following incubation, the biofilms were treated with 2xMBC, MBC, and MIC of extracts for an additional 24 h at 37 °C after the wells had been cleaned twice with sterile PBS. Following two PBS washes for each well, methanol was used to fix the biofilms, and the plate was allowed to air dry. For thirty minutes, the biofilm was dyed with 0.1% crystal violet (Bio-Merieux, Craponne, France). Following the removal of crystal violet, the wells were cleaned under tap water, allowed to air dry, and then the bound dye was dissolved using 100 μL of 96% ethanol (Zorka, Bezdan, Serbia). The absorbance was read at 620 nm on a Multiskan^TM^ FC Microplate Photometer (Thermo Scientific^TM^, Waltham, MA USA), and the percentage of biofilm destruction was calculated by the following formula:

[(A620 control − A620 sample)/A620 control] × 100

### 3.7. Congo Red Test

Following Ivanov et al.’s procedure, the Congo red binding test was used to evaluate the impact of extracts on the synthesis of exopolysaccharides (EPS), which are crucial parts of biofilms [48]. For 24 h, *S. aureus* bacterial biofilms were grown at 37 °C with the extract (½ MIC). PBS was used to wash adherent cells after planktonic cells were eliminated. For 30 min in the dark, 150 μL of 1% Congo red solution (SigmaAldrich, Darmstadt, Germany) was used to stain the wells. After the wells were emptied, 100 μL of DMSO was added to dissolve the bound dye. Through the use of the Sunrise^TM^ ELISA reader (Tecan Group Ltd., Männedorf, Switzerland) absorbance was measured in a microtiter plate at 490 nm. Using the following formula, the % inhibition of EPS generation was determined:

% inhibition = [(A490 control − A490 sample)/A490 control] × 100

where

A490 control = absorbance of the untreated biofilm;

A490 sample = absorbance of the sample treated with the extract.

### 3.8. eDNA Test

This test quantifies extracellular DNA as an essential component of biofilms [49]. The *S. aureus* strain was cultivated for 24 h at 37 °C in 96-well plates (Sarstedt, Nümbrecht, Germany) using TSB medium supplemented with 2% glucose and extract at concentrations specified in the Congo red assay. Planktonic cells were eliminated after incubation, and PBS was used to wash the wells. After adding TE buffer (Tris-EDTA buffer solution) to each well, the contents were pipetted forcefully. After being transferred to 1.5 mL tubes, the samples were centrifuged for 10 min at 10,000× *g*. The pellet was vortexed in TE buffer to suspend it after the supernatant was removed. The supernatant was centrifuged at 10,000× *g* for 15 min, and its absorbance was measured at 260 nm. The percentage inhibition of eDNA relative to the untreated control was calculated using the following formula:

% inhibition = [(A260 control − A260 sample)/A260 control] × 100

where

A260 control = absorbance of the untreated biofilm;

A260 sample = absorbance of the sample treated with the extract.

### 3.9. Analysis of Cytotoxicity of the Extract

The cytotoxicity of the extracts was tested on a spontaneously immortalized human keratinocyte cell line (HaCaT—AddexBio T0020001), MCF7 (breast ad-denocarcinoma—ATCC HTB-22^TM^), HeLa (cervical cancer—ATCC CCL-2^TM^), CaCo2 (colorectal cancer—ATCC HTB-37^TM^), and HepG2 (liver cancer—ATCC HB-8065^TM^) using the crystal violet assay as described previously [50]. To reach a final concentration of 8 mg/mL, the extracts were dissolved in phosphate-buffered saline (PBS). The findings were presented as an IC_50_ value, which, when compared to an untreated control, indicated a 50% reduction of cell growth. Three separate tests were conducted, with each concentration of the extracts being tested in triplicate. The solvent was used as a negative control, while ellipticine was used as a positive control.

### 3.10. Effects of the Extract on the Invasion Capacities of S. aureus in HaCaT Cells

Assessment of the ability of extracts to reduce invasion capacities of *S. aureus* towards HaCaT cells was determined as described in Ahmed et al. [51] with some modifications.

HaCaT cells were cultured till confluence in 24-well plates with an adhesive bottom. Following the removal of the medium, the cells were given fresh DMEM containing the extract but without FBS. Following a 15 min incubation period at 37 °C, 100 µL of *S. aureus* culture (10^8^ CFU/mL) was added to the cells. The cells were then incubated for 2 h at 37 °C to test the invasion capacity, giving the bacterial cells enough time to infiltrate HaCaT. Gentamicin (300 µg/mL) was then added to the cells for 1 h to kill the adherent bacterial cells. The cells were then lysed for 30 min at 37 °C using 1 mL of 1% (*v*/*v*) Tween-20 (SigmaAldrich, Germany) after being rinsed three times with DMEM devoid of FBS. The *S. aureus* suspension in each well was then diluted and seeded onto Trypton Soy Agar plates. The number of CFU was determined after an 18 h incubation period at 37 °C.

### 3.11. Molecular Docking

In this study, the proteins and enzymes were sourced from the Protein Data Bank (PDB). For detailed descriptions of the standard enzymes and relevant proteins, please refer to Table S1. However, in this specific research, the proteins were selected based on their associations with the organisms and cancer types under investigation, namely *L. monocytogenes* [52,53], *B. cereus* [54,55], *P. aeruginosa* [54,56,57,58], *S. aureus*, *E. coli* [59,60], colon adenocarcinoma [54,61], and lung adenocarcinoma [54]. The selected proteins were analyzed for their interactions with the fatty acids linolenic acid, palmitic acid, oleic acid, palmitoleic acid, and linoleic acid. In order to prepare the protein structures for molecular docking investigations, the co-crystallized ligands, cofactors, and water molecules have been removed from them using BIOVIA Discovery Studio Visualizer V4.5 [62,63,64,65,66]. The ligands were acquired from the PubChem database and optimized using OpenBabel V3.1.1. MGL Tools V1.5.6 was then used to create the protein and enzyme structures for molecular docking. POCASA V1.1 was used to identify active binding sites, or inhibitor binding sites reported in the literature were used as a basis (Table S2) [54]. The proteins were re-docked with their corresponding ligands, and the RMSD values were computed to confirm the correctness of the docking procedure. AutoDock Vina V1.1.2 was used for molecular docking. The methods described by Trott and Olson [67] were used to define the grid boxes. Following docking, PLIP was used to study the protein–ligand interactions and assess important characteristics including hydrogen bonds. The accuracy and dependability of the docking results were guaranteed by this analysis.

### 3.12. Calculation of MM/PBSA Free Energy to Determine Ligand-Binding Affinity

This study employed the gmx_MMPBSA tool (https://valdes-tresanco-ms.github.io/gmx_MMPBSA/dev/getting-started/ (accessed on 13 April 2025)) to evaluate the stability and calculate the binding free energy of various molecular complexes. To evaluate the stability of the aforementioned complexes, 100 ns molecular dynamics (MD) simulations were conducted. The complexes palmitic acid_PrfA, oleic acid_LasR, palmitoleic acid_EGFR, and linolenic acid_AKT-1 were subjected to molecular dynamics simulations. Based on these simulations, the complexes demonstrating the highest stability were identified and selected for further investigation [68,69].

### 3.13. Molecular Dynamics Simulation Setup and Analysis Parameters

In this study, we performed molecular dynamics simulations complexes to investigate their stability and potential therapeutic interactions. System preparation was carried out using the CHARMM-GUI platform, specifically employing the Solution Builder tool as described by Jo et al. [70]. For protein parametrization, we selected the CHARMM36m force field in accordance with the methodologies outlined by Yagi et al. [66] and Maier et al. [71]. TIP3P water molecules were used to solvate each complex, guaranteeing that the protein and the simulation box’s boundaries were at least 10 Å apart. Counterions were introduced to reach a NaCl concentration of 0.15 M in order to neutralize the systems and replicate physiological circumstances. The Particle Mesh Ewald (PME) method was used to determine electrostatic interactions, and a cutoff distance of 12 Å was used for van der Waals interactions. With a time step of 2 fs, we used the LINCS algorithm to restrict bonds involving hydrogen atoms. The steepest descent method was used to minimize energy until the maximum force was less than 1000 kJ/mol/nm. To guarantee thermodynamic stability, the systems were then equilibrated under both NVT and NPT ensembles at 303 K for 1 ns each. The GROMACS 2023.2 program was then used to run production simulations for 100 ns. In order to assess the stability and dynamic behavior of the complexes, we examined parameters including RMSD, RMSF, SASA, minimum distance, and hydrogen bond interactions during the simulations. We were better able to comprehend the complexes’ interaction processes and therapeutic potential thanks to these analyses.

## 4. Conclusions

There is an urgent need to develop novel antimicrobial treatments for *Staphylococcus aureus*, a major pathogen responsible for both hospital-associated and community-acquired infections. This bacterium poses significant challenges due to its ability to form biofilms, which not only protect it from antibiotics but also contribute to chronic infections. Our study provides valuable insights into the antibacterial and *S.aureus* antibiofilm properties of purslane extract, particularly its fatty acid components. Also, our results demonstrated that the inhibition of extracellular DNA (eDNA) production within the biofilm matrix may play a key role in the potent antibiofilm activity of tested extracts, positioning it as a promising candidate for future therapeutic development. Building on these results and in line with recent investigations, more investigation is required to examine the molecular mechanisms behind purslane extract’s interference with biofilm formation and maintenance. The optimization of extract formulations, evaluation of synergistic effects with traditional antibiotics, and in vivo studies to confirm efficacy should be the main goals of future research. Using cutting-edge analytical methods like multi-omics and high-resolution imaging may provide fresh perspectives on the bioactive elements and how they interact within bacterial communities, which could help direct the creation of integrated antimicrobial strategies that more successfully fight infections linked to biofilms.

## Figures and Tables

**Table 1:** Fatty acid composition of purslane samples from Greece and Serbia (mean ± SD).

	Content (%)	
	Sample from Serbia	Sample from Greece
C6:0	0.17 ± 0.00	0.21 ± 0.00
C8:0	0.25 ± 0.00	0.23 ± 0.00
C10:0	5.43 ± 0.00	6.21 ± 0.00
C12:0	0.01 ± 0.00	0.04 ± 0.00
C13:0	0.08 ± 0.00	0.06 ± 0.00
C14:0	0.22 ± 0.01	0.21 ± 0.01
C15:0	0.18 ± 0.00	0.16 ± 0.00
C16:0	13.87 ± 0.23	14.07 ± 0.07
C16:1	6.89 ± 0.01	0.64 ± 0.01
C17:0	0.37 ± 0.01	0.37 ± 0.03
C18:0	4.41 ± 0.02	7.43 ± 0.03
C18:1n9	9.72 ± 0.04	10.50 ± 0.04
C18:2n6	31.42 ±0.07	34.52 ± 0.08
C18:3n3	23.34 ± 0.02	23.22 ± 0.01
C20:0	0.22 ± 0.00	0.12 ± 0.00
C20:1	0.38 ± 0.01	0.33 ± 0.01
C20:2	0.14 ± 0.00	0.12 ± 0.00
C20:3n3 + C21:0	0.36 ± 0.00	0.03 ± 0.00
C20:5n3	0.25 ± 0.01	0.01 ± 0.01
C22:0	1.01 ± 0.03	1.02 ± 0.03
C22:1n9	0.11 ± 0.00	0.11 ± 0.00
C23:0	0.19 ± 0.03	0.10 ± 0.03
C24:0	0.89 ± 0.09	0.21 ± 0.03
C24:1	0.09 ± 0.03	0.08 ± 0.03
Total SFA (% of total FA)	27.30 ± 0.07	30.44 ± 0.08
Total MUFA (% of total FA)	17.19 ± 0.01	11.66 ± 0.01
Total PUFA (% of total FA)	55.51 ± 0.07	57.90± 0.07

**Table 2:** Minimal inhibitory (MIC), bactericidal (MBC), and fungicidal (MFC) concentrations of purslane extracts from Greece and Serbia. Results are expressed in mg/mL.

		*S.a.*	*B.c.*	*L.m.*	*M.f.*	*P.a.*	*E.c.*	*S.t.*	*En.cl.*
Greece	MIC	0.59	1.18	4.74	2.38	0.59	2.38	2.38	1.18
	MBC	1.18	2.38	9.49	4.74	1.18	4.74	4.74	2.38
Serbia	MIC	1.18	2.38	9.49	2.38	1.18	4.74	4.74	1.18
	MBC	1.18	4.74	9.49	4.74	1.18	4.74	4.74	2.38
Streptomycin	MIC	0.25	0.05	0.15	0.125	0.05	0.05	0.05	0.05
	MBC	0.50	0.10	0.30	0.25	0.10	0.10	0.10	0.10
Ampicillin	MIC	0.10	0.10	0.15	0.10	0.10	0.30	0.15	0.15
	MBC	0.15	0.15	0.30	0.15	0.20	0.50	0.20	0.20
		***A.f.***	***A.v.***	***A.o.***	***A.n.***	***T.v.***	***P.f.***	***P.o.***	***P.c.***
Greece	MIC	2.38	0.025	1.19	2.38	2.38	2.38	1.19	2.38
	MFC	18.96	0.95	4.74	9.49	2.38	4.74	4.74	4.47
Serbia	MIC	9.49	0.95	2.38	4.74	2.38	2.38	1.19	2.38
	MBC	18.96	1.19	4.74	9.49	4.74	4.74	4.74	9.49
Ketoconazole	MIC	0.20	0.20	0.15	0.20	0.20	2.50	0.20	0.25
	MFC	0.50	0.50	0.20	0.50	0.30	3.50	0.50	0.50
Bifonazole	MIC	0.15	0.10	0.15	0.15	0.10	0.20	0.20	0.25
	MFC	0.20	0.20	0.20	0.20	0.20	0.25	0.25	0.50

***S.a.***—*Staphylococcus aureus*; ***B.c.***—*Bacillus cereus*; ***M.f.***—*Micrococcus flavus*; ***L.m.***—*Listeria monocytogenes*; ***P.a.***—*Pseudomonas aeruginosa*; ***S.t.***—*Salmonella typhimurium*; ***E.c.***—*Escherichia coli*; ***En.cl.***—*Enterobacter cloacae*; ***A.f.***—*Aspergillus fumigatus*; ***A.v.***—*Aspergillus versicolor*; ***A.o.***—*Aspergillus ochraceus*; ***A.n.***—*Aspergillus niger*; ***T.v.***—*Trichoderma viride*; ***P.f.***—*Penicillium funiculosum*; ***P.o.***—*Penicillium ochrochloron*; ***P.c.***—*Penicillium verrucosum* var. *cyclopium*.

**Table 3:** Antibiofilm activity of purslane extracts (%).

Sample	MIC	½ MIC	¼ MIC
Greece	87.91 ± 4.38	75.42 ± 5.58	41.22 ± 6.44
Serbia	89.54 ± 7.66	81.21 ± 9.11	51.47 ± 2.99

**Table 4:** Effects of purslane extracts on *S. aureus* exopolysaccharide production inhibition (%).

Sample	½ MIC
Greece	56.11 ± 2.93
Serbia	41.71 ± 7.17

**Table 5:** Extracellular DNA production inhibition by purslane extracts from Serbia and Greece (%).

Sample	½ MIC
Greece	76.61 ± 4.24
Serbia	62.33 ± 5.65

**Table 6:** IC_50_ values for tested purslane extracts from Serbia and Greece against various cell lines.

Cell Lines	Greece IC_50_ (µg/mL)	Serbia IC_50_ (µg/mL)	Ellipticine IC_50_ (µg/mL)
HaCaT	>400	>400	>400
MCF7	397.31 ± 26.41	374.33 ± 18.39	1.21 ± 0.01
HeLa	354.79 ± 20.57	361.96 ± 18.36	1.02 ± 0.02
CaCo2	198.44 ± 11.15	178.17 ± 9.33	1.42 ± 0.02
HepG2	292.37 ± 21.33	272.36 ± 17.29	1.05 ± 0.01

**Table 7:** The docking score (kcal/mol) and interacting residues of the enzyme and protein.

Compound	Protein Name	PDB ID	Binding Energy	RMSD	Interaction		Binding Site
					Type	Number	
Linolenic acid	TubR	6AHT	−5	nd	Hbond	2	ALA A:70; THR A:75
Linolenic acid	Transpeptidase	5TW8	−6	nd	Hbond	2	GLY A:261; SER A:262
Linoleic acid	Transpeptidase	5TW8	−5	13.4	Hbond	3	ASN A:72; GLY A:181; SER A:263
Linolenic acid	PrfA	6EXL	−7	8.1	Hbond	2	TYR A:126; TYR A:126
Palmitic acid	PrfA	6EXL	−7	1.7	Hbond	2	TYR A:126; GLN A:146
Oleic acid	PrfA	6EXL	−7	5.3	Hbond	2	LYS A:64; LYS A:64
Palmitoleic acid	PrfA	6EXL	−7	5.6	Hbond	1	LYS A:64
Linoleic acid	PrfA	6EXL	−7	1.9	Hbond	1	TYR A:126
Linolenic acid	PI3K	4XE0	−7	7.1	Hbond	3	GLN A:610; LEU A:613; GLN A:792
Palmitic acid	PI3K	4XE0	−6	8.3	Hbond	3	LEU A:612; LEU A:613; GLN A:792
Oleic acid	PI3K	4XE0	−6	8.6	Hbond	1	PRO A:812
Linoleic acid	PI3K	4XE0	−6	6.6	Hbond	1	CYS A:815
Oleic acid	MurE	4C13	−5	10.0	Hbond	2	TYR A:351; TYR A:351
Linoleic acid	MurE	4C13	−5	nd	Hbond	5	GLY A:113; LYS A:114; THR A:115; THR A:115; THR A:137
Linolenic acid	MurE	1E8C	−5	nd	Hbond	1	GLY B:304
Linolenic acid	MDM2	4WT2	−6	1.1	Hbond	1	THR A:16
Palmitic acid	MDM2	4WT2	−5	6.3	Hbond	1	VAL A:14
Oleic acid	MDM2	4WT2	−6	9.4	Hbond	1	TYR A:100
Palmitoleic acid	MDM2	4WT2	−6	7.1	Hbond	1	VAL A:93
Linoleic acid	MDM2	4WT2	−7	8.6	Hbond	1	GLN A:59
Oleic acid	LasR	2UV0	−8	1.7	Hbond	2	TYR E:56; TYR E:93
Linolenic acid	InlA	1O6T	−6	41.9	Hbond	4	SER A:429; SER A:429; SER A:429; SER A:429
Oleic acid	InlA	1O6T	−5	nd	Hbond	5	ASP A:265; ILE A:266; ASN A:287; ASN A:287; ASN A:287
Linoleic acid	InlA	1O6T	−5	29.6	Hbond	3	GLU A:326; GLN A:328; GLN A:328
Linolenic acid	EGFR	1M17	−5	8.9	Hbond	2	MET A:769; MET A:769
Oleic acid	EGFR	1M17	−5	nd	Hbond	2	GLU A:738; THR A:766
Palmitoleic acid	EGFR	1M17	−5	1.9	Hbond	4	GLU A:738; ASP A:831; ASP A:831; ASP A:831
Linoleic acid	EGFR	1M17	−5	1.6	Hbond	1	THR A:766
Palmitoleic acid	DNA Gyrase B	1KZN	−5	nd	Hbond	2	THR A:165; VAL A:167
Oleic acid	Cyclin D1	2W99	−5	1.9	Hbond	1	GLN A:183
Linolenic acid	CDKN1A	5E0U	−5	nd	Hbond	2	GLN A:131; TYR A:133
Palmitic acid	CDKN1A	5E0U	−5	nd	Hbond	1	HIS A:44
Oleic acid	CDKN1A	5E0U	−5	nd	Hbond	2	GLN A:131; TYR A:133
Linolenic acid	CDK2	6GUE	−6	3.2	Hbond	1	VAL A:163
Palmitic acid	CDK2	6GUE	−6	3.6	Hbond	1	VAL A:163
Oleic acid	CDK2	6GUE	−6	3.2	Hbond	1	TYR A:180
Palmitoleic acid	CDK2	6GUE	−6	7.8	Hbond	1	TYR A:180
Linoleic acid	CDK2	6GUE	−6	2.4	Hbond	1	VAL A:163
Linolenic acid	AKT-1	4VG1	−5	1.7	Hbond	2	PHE A:293; GLY A:294
Linoleic acid	AKT-1	4VG1	−6	6.0	Hbond	1	GLU A:191

## Data Availability

Data are contained within the article and the Supplementary Materials.

## Supplementary Materials

The following supporting information can be downloaded at: https://www.mdpi.com/article/10.3390/ph18040587/s1, Table S1. Common names of fatty acids found in purslane. Table S2. Relevant protein and enzyme target coordinates of the docking box. Table S3. Relevant protein and enzyme result of the docking scores. Table S4. Selected protein-ligand complexes for MM/PBSA binding free energy analysis based on molecular dynamics simulations. Reference [72] is cited in the Supplementary Materials.

## References

[1] Sultana A., Raheman K. (2013). Portulaca oleracea Linn: A global panacea with ethnomedicinal and pharmacological potential. Int. J. Pharm. Pharm. Sci..

[2] Iranshahy M., Javadi B., Iranshahi M., Jahanbakhsh S.P., Mahyari S., Hassani F.V., Karimi G. (2017). A review of traditional uses, phytochemistry and pharmacology of *Portulaca oleracea* L. J. Ethnopharmacol..

[3] Holm L.G., Plucknett D.L., Pancho J.V., Herberger J.P. (1977). The World’s Worst Weeds. Distribution and Biology.

[4] Uddin M.K., Juraimi A.S., Anwar F., Hossain M.A., Alam M.A. (2012). Effect of salinity on proximate mineral composition of purslane (‘*Portulca oleracea*’ L.). Aust. J. Crop Sci..

[5] Ocampo G., Columbus J.T. (2012). Molecular phylogenetics, historical biogeography, and chromosome number evolution of *Portulaca* (Portulacaceae). Mol. Phylogenetics Evol..

[6] Samir D., Sara C., Widad A. (2022). The effects of aqueous leaf extract of *Portulaca oleracea* on haemato-biochemical and histopathological changes induced by sub-chronic aluminium toxicity in male wistar rats. Pharmacol. Res.-Mod. Chin. Med..

[7] Abd El-Hack M.E., Alabdali A.Y., Aldhalmi A.K., Reda F.M., Bassiony S.S., Selim S., El-Saadony M.T., Alagawany M. (2022). Impacts of Purslane (*Portulaca oleracea*) extract supplementation on growing Japanese quails’ growth, carcass traits, blood indices, nutrients digestibility and gut microbiota. Poult. Sci..

[8] Zhou Y.-X., Xin H.-L., Rahman K., Wang S.-J., Peng C., Zhang H. (2015). *Portulaca oleracea* L.: A review of phytochemistry and pharmacological effects. BioMed Res. Int..

[9] Mohamed A.I., Hussein A.S. (1994). Chemical composition of purslane (*Portulaca oleracea*). Plant Foods Hum. Nutr..

[10] Naeem F., Khan S.H. (2013). Purslane (*Portulaca oleracea* L.) as phytogenic substance—A review. J. Herbs Spices Med. Plants.

[11] Zouine N., Ghachtouli N.E., Abed S.E., Koraichi S.I. (2024). A comprehensive review on medicinal plant extracts as antibacterial agents: Factors, mechanism insights and future prospects. Sci. Afr..

[12] Puca V., Marulli R.Z., Grande R., Vitale I., Niro A., Molinaro G., Prezioso S., Muraro R., Di Giovanni P. (2021). Microbial Species Isolated from Infected Wounds and Antimicrobial Resistance Analysis: Data Emerging from a Three-Years Retrospective Study. Antibiotics.

[13] Yang S., Qiao J., Zhang M., Kwok L.-Y., Matijašić B.B., Zhang H., Zhang W. (2024). Prevention and treatment of antibiotics-associated adverse effects through the use of probiotics: A review. J. Adv. Res..

[14] Zasheva D., Mladenov P., Zapryanova S., Gospodinova Z., Georgieva M., Alexandar I., Velinov V., Djilianov D., Moyankova D., Simova-Stoilova L. (2024). Cytotoxic Effects of Plant Secondary Metabolites and Naturally Occurring Bioactive Peptides on Breast Cancer Model Systems: Molecular Mechanisms. Molecules.

[15] Simopoulos A.P., Norman H.A., Gillaspy J.E., Duke J.A. (1992). Common purslane: A source of omega-3 fatty acids and antioxidants. J. Am. Coll. Nutr..

[16] Oliveira I., Valentão P., Lopes R., Andrade P.B., Bento A., Pereira J.A. (2009). Phytochemical characterization and radical scavenging activity of *Portulaca oleraceae* L. leaves and stems. Microchem. J..

[17] Simopoulos A.P. (2008). The importance of the omega-6/omega-3 fatty acid ratio in cardiovascular disease and other chronic diseases. Exp. Biol. Med..

[18] Othman A.S. (2017). Bactericidal efficacy of omega-3 fatty acids and esters present in *Moringa oleifera* and *Portulaca oleracea* fixed oils against oral and gastro enteric bacteria. Int. J. Pharmacol..

[19] Tleubayeva M.I., Datkhayev U.M., Alimzhanova M., Ishmuratova M.Y., Korotetskaya N.V., Abdullabekova R.M., Flisyuk E.V., Gemejiyeva N.G. (2021). Component Composition and Antimicrobial Activity of CO_2_ Extract of *Portulaca oleracea*, Growing in the Territory of Kazakhstan. Sci. World J..

[20] Banerjee G., Mukherjee A. (2002). Biological activity of a common weed-*Portulaca oleracea* L. II. Antifungal activity. Acta Bot. Hung..

[21] Zheng C.J., Yoo J.-S., Lee T.-G., Cho H.-Y., Kim Y.-H., Kim W.-G. (2005). Fatty acid synthesis is a target for antibacterial activity of unsaturated fatty acids. FEBS Lett..

[22] Kelsey J., Bayles K.W., Shafii B., McGuire M. (2006). Fatty acids and monoacylglycerols inhibit growth of *Staphylococcus aureus*. Lipids.

[23] Fung K., Han Q., Ip M., Yang X., Lau C.B., Chan B.C. (2017). Synergists from *Portulaca oleracea* with macrolides against methicillin-resistant *Staphylococcus aureus* and related mechanism. Hong Kong Med. J..

[24] Nguyen M.T., Hanzelmann D., Härtner T., Peschel A., Götz F. (2016). Skin-specific unsaturated fatty acids boost the *Staphylococcus aureus* innate immune response. Infect. Immun..

[25] Cho J.-A., Roh Y.J., Son H.R., Choi H., Lee J.-W., Kim S.J., Lee C.-H. (2022). Assessment of the biofilm-forming ability on solid surfaces of periprosthetic infection-associated pathogens. Sci. Rep..

[26] Valle J., Echeverz M., Lasa I. (2019). σ(B) Inhibits Poly-N-Acetylglucosamine Exopolysaccharide Synthesis and Biofilm Formation in *Staphylococcus aureus*. J. Bacteriol..

[27] Ma S., Liu J., Feng L., Wang C., Ren J., Yang C., Dai Y., Cui G., Zhang K., Li C. (2013). Study on the antimicrobial effects of aqueous extracts from *Portulaca oleracea* L. J. Pure Appl. Microbiol..

[28] Yuyama K.T., Rohde M., Molinari G., Stadler M., Abraham W.-R. (2020). Unsaturated fatty acids control biofilm formation of Staphylococcus aureus and other gram-positive bacteria. Antibiotics.

[29] Kim H.-S., Ham S.-Y., Jang Y., Sun P.-F., Park J.-H., Hoon Lee J., Park H.-D. (2019). Linoleic acid, a plant fatty acid, controls membrane biofouling via inhibition of biofilm formation. Fuel.

[30] Samrot A.V., Abubakar Mohamed A., Faradjeva E., Si Jie L., Hooi Sze C., Arif A., Chuan Sean T., Norbert Michael E., Yeok Mun C., Xiao Qi N. (2021). Mechanisms and impact of biofilms and targeting of biofilms using bioactive compounds—A review. Medicina.

[31] Mombeshora M., Chi G.F., Mukanganyama S. (2021). Antibiofilm Activity of Extract and a Compound Isolated from *Triumfetta welwitschii* against *Pseudomonas aeruginosa*. Biochem. Res. Int..

[32] Okshevsky M., Regina V.R., Meyer R.L. (2015). Extracellular DNA as a target for biofilm control. Curr. Opin. Biotechnol..

[33] Sugimoto S., Sato F., Miyakawa R., Chiba A., Onodera S., Hori S., Mizunoe Y. (2018). Broad impact of extracellular DNA on biofilm formation by clinically isolated Methicillin-resistant and-sensitive strains of *Staphylococcus aureus*. Sci. Rep..

[34] Wei M., Wang P., Li T., Wang Q., Su M., Gu L., Wang S. (2023). Antimicrobial and antibiofilm effects of essential fatty acids against clinically isolated vancomycin-resistant *Enterococcus faecium*. Front. Cell. Infect. Microbiol..

[35] Shah S., Zubair M. (2019). Membrane Disruption and Biofilm Inhibition by Fatty Acids in *Pseudomonas aeruginosa*. J. Microbiol..

[36] Knap K., Kwiecień K., Ochońska D., Reczyńska-Kolman K., Pamuła E., Brzychczy-Włoch M. (2024). Synergistic effect of antibiotics, α-linolenic acid and solvent type against Staphylococcus aureus biofilm formation. Pharmacol. Rep..

[37] Al-Sheddi E.S., Farshori N.N., Al-Oqail M.M., Musarrat J., Al-Khedhairy A.A., Siddiqui M.A. (2015). *Portulaca oleracea* Seed Oil Exerts Cytotoxic Effects on Human Liver Cancer (HepG2) and Human Lung Cancer (A-549) Cell Lines. Asian Pac. J. Cancer Prev..

[38] Asif M. (2011). Health effects of omega-3,6,9 fatty acids: *Perilla frutescens* is a good example of plant oils. Orient. Pharm. Exp. Med..

[39] Petropoulos S.A., Fernandes Â., Dias M.I., Vasilakoglou I.B., Petrotos K., Barros L., Ferreira I.C.F.R. (2019). Nutritional Value, Chemical Composition and Cytotoxic Properties of Common Purslane (*Portulaca oleracea* L.) in Relation to Harvesting Stage and Plant Part. Antioxidants.

[40] Scheim D.E. (2009). Cytotoxicity of unsaturated fatty acids in fresh human tumor explants: Concentration thresholds and implications for clinical efficacy. Lipids Health Dis..

[41] Banfi C., Risé P., Mussoni L., Galli C., Tremoli E. (1997). Linoleic acid enhances the secretion of plasminogen activator inhibitor type 1 by HepG2 cells. J. Lipid Res..

[42] Nuzzo I., Sanges M., Folgore A., Carratelli C.R. (2000). Apoptosis of human keratinocytes after bacterial invasion. FEMS Immunol. Med. Microbiol..

[43] Kenny J.G., Ward D., Josefsson E., Jonsson I.-M., Hinds J., Rees H.H., Lindsay J.A., Tarkowski A., Horsburgh M.J. (2009). The Staphylococcus aureus Response to Unsaturated Long Chain Free Fatty Acids: Survival Mechanisms and Virulence Implications. PLoS ONE.

[44] Pereira C., Barros L., Carvalho A.M., Ferreira I.C. (2013). Use of UFLC-PDA for the analysis of organic acids in thirty-five species of food and medicinal plants. Food Anal. Methods.

[45] Soković M., Glamočlija J., Marin P.D., Brkić D., Van Griensven L.J. (2010). Antibacterial effects of the essential oils of commonly consumed medicinal herbs using an in vitro model. Molecules.

[46] Soković M., Van Griensven L.J. (2006). Antimicrobial activity of essential oils and their components against the three major pathogens of the cultivated button mushroom, *Agaricus bisporus*. Eur. J. Plant Pathol..

[47] Smiljković M., Dias M.I., Stojković D., Barros L., Bukvički D., Ferreira I.C., Soković M. (2018). Characterization of phenolic compounds in tincture of edible *Nepeta nuda*: Development of antimicrobial mouthwash. Food Funct..

[48] Ivanov M., Kostić M., Stojković D., Soković M. (2022). Rosmarinic acid–modes of antimicrobial and antibiofilm activities of a common plant polyphenol. S. Afr. J. Bot..

[49] Carević T., Kolarević S., Kolarević M.K., Nestorović N., Novović K., Nikolić B., Ivanov M. (2024). Citrus flavonoids diosmin, myricetin and neohesperidin as inhibitors of *Pseudomonas aeruginosa*: Evidence from antibiofilm, gene expression and in vivo analysis. Biomed. Pharmacother..

[50] Stojković D., Gašić U., Drakulić D., Zengin G., Stevanović M., Rajčević N., Soković M. (2021). Chemical profiling, antimicrobial, anti-enzymatic, and cytotoxic properties of *Phlomis fruticosa* L. J. Pharm. Biomed. Anal..

[51] Ahmed G.F., Elkhatib W.F., Noreddin A.M. (2014). Inhibition of *Pseudomonas aeruginosa* PAO1 adhesion to and invasion of A549 lung epithelial cells by natural extracts. J. Infect. Public Health.

[52] Deepasree K., Subhashree V. (2023). Molecular docking and dynamic simulation studies of terpenoid compounds against phosphatidylinositol-specific phospholipase C from *Listeria monocytogenes*. Inform. Med. Unlocked.

[53] Venugopal S. (2024). Molecular docking and molecular dynamic simulation studies to identify potential terpenes against Internalin A protein of *Listeria monocytogenes*. Front. Bioinform..

[54] Yu J., Zhou Y., Tanaka I., Yao M. (2010). Roll: A new algorithm for the detection of protein pockets and cavities with a rolling probe sphere. Bioinformatics.

[55] Zhang Y., Qiao P., Li S., Feng X., Bian L. (2017). Molecular recognition and binding of beta-lactamase II from Bacillus cereus with penicillin V and sulbactam by spectroscopic analysis in combination with docking simulation. Luminescence.

[56] Baburam S., Ramasamy S., Shanmugam G., Mathanmohun M. (2022). Quorum sensing inhibitory potential and molecular docking studies of *Phyllanthus emblica* phytochemicals against Pseudomonas aeruginosa. Appl. Biochem. Biotechnol..

[57] Malathi K., Ramaiah S. (2016). Molecular docking and molecular dynamics studies to identify potential OXA-10 extended spectrum β-lactamase non-hydrolysing inhibitors for *Pseudomonas aeruginosa*. Cell Biochem. Biophys..

[58] Zuo K., Liang L., Du W., Sun X., Liu W., Gou X., Wan H., Hu J. (2017). 3D-QSAR, molecular docking and molecular dynamics simulation of *Pseudomonas aeruginosa* LpxC inhibitors. Int. J. Mol. Sci..

[59] Hetmann M., Langner C., Durmaz V., Cespugli M., Köchl K., Krassnigg A., Blaschitz K., Groiss S., Loibner M., Ruau D. (2023). Identification and validation of fusidic acid and flufenamic acid as inhibitors of SARS-CoV-2 replication using DrugSolver CavitomiX. Sci. Rep..

[60] Saqallah F.G., Hamed W.M., Talib W.H., Dianita R., Wahab H.A. (2022). Antimicrobial activity and molecular docking screening of bioactive components of *Antirrhinum majus* (snapdragon) aerial parts. Heliyon.

[61] Sun R., Zhu J., Sun K., Gao L., Zheng B., Shi J. (2023). Strontium Ranelate Ameliorates Intervertebral Disc Degeneration via Regulating TGF-β1/NF-κB Axis. Int. J. Med. Sci..

[62] Cetiz M.V., Isah M., Ak G., Bakar K., Himidi A.A., Mohamed A., Glamočlija J., Nikolić F., Gašic U., Cespedes-Acuna C.L. (2024). Exploring of Chemical Profile and Biological Activities of Three Ocimum Species from Comoros Islands: A Combination of In Vitro and In Silico Insights. Cell Biochem. Funct..

[63] Cusumano G., Flores G.A., Cetiz M.V., Kurt U., Ak G., Saka E., Aly S.H., Eldahshan O.A., Singab A.N., Zengin G. (2024). Small Steps to the Big Picture for Health-Promoting Applications Through the Use of Chickweed (*Stellaria media*): In Vitro, In Silico, and Pharmacological Network Approaches. Food Sci. Nutr..

[64] Duran T., Peron G., Zancato M., Zengin G., Cetiz M.V., Bouyahya A., Ahmed S., Yildiztugay E., Dall’Acqua S., Kljakić A.C. (2024). Harnessing the chemical composition and anti-oxidant, anti-enzymatic, and anti-cancer activities of two Corydalis species (*C. erdelii* and *C. solida*) by using in vitro and in silico analysis. Food Biosci..

[65] Kurt-Celep I., Nilofar, Cetiz M.V., Zheleva-Dimitrova D., Gevrenova R., Celep E., Sinan K.I., Yildiztugay E., Ferrante C., Zengin G. (2025). From small-scale studies to an encompassing view: Inhibiting inflammation and clinically relevant enzymes with various extracts of *Primula vulgaris* using in vitro and in silico techniques. Food Front..

[66] Yagi S., Zengin G., Eldahshan O.A., Singab A.N.B., Selvi S., Cetiz M.V., Rodrigues M.J., Custodio L., Dall’Acqua S., Elhawary E.A. (2024). Functional constituents of *Colchicum lingulatum* Boiss. & Spruner subsp. *Rigescens* K. Perss. Extracts and their biological activities with different perspectives. Food Biosci..

[67] Trott O., Olson A.J. (2010). AutoDock Vina: Improving the speed and accuracy of docking with a new scoring function, efficient optimization, and multithreading. J. Comput. Chem..

[68] Miller B.R., McGee T.D., Swails J.M., Homeyer N., Gohlke H., Roitberg A.E. (2012). MMPBSA. py: An efficient program for end-state free energy calculations. J. Chem. Theory Comput..

[69] Valdés-Tresanco M.S., Valdés-Tresanco M.E., Valiente P.A., Moreno E. (2021). gmx_MMPBSA: A new tool to perform end-state free energy calculations with GROMACS. J. Chem. Theory Comput..

[70] Jo S., Kim T., Iyer V.G., Im W. (2008). CHARMM-GUI: A web-based graphical user interface for CHARMM. J. Comput. Chem..

[71] Maier J.A., Martinez C., Kasavajhala K., Wickstrom L., Hauser K.E., Simmerling C. (2015). ff14SB: Improving the accuracy of protein side chain and backbone parameters from ff99SB. J. Chem. Theory Comput..

[72] Karthick K., Abishek K., Angel Jemima E. (2024). In Silico Study, Protein Kinase Inhibition and Molecular Docking Study of Benzimidazole Derivatives. Bioinform. Biol. Insights.

