# Supplementary material for: Lipophilic Extracts of Portulaca oleracea L.: Analysis of Bioactive Fatty Acids Targeting Microbial and Cancer Pathways
Source: Pharmaceuticals (Basel). 2025 Apr 17;18(4):587. doi: 10.3390/ph18040587 (PMC12030143; doi:10.3390/ph18040587)
Supplement: Supplementary file 1 [file pharmaceuticals-18-00587-s001.zip › pharmaceuticals-3582089-supplementary.pdf]

Supplementary Tables

Table S1. Common names of fatty acids found in purslane

	Name
C6:0	Caproic acid
C8:0	Caprylic acid
C10:0	Capric acid
C12:0	Lauric acid
C13:0	Tridecanoic acid
C14:0	Myristic acid
C15:0	Pentadecanoic acid
C16:0	Palmitic acid
C16:1	Palmitoleic acid
C17:0	Heptadecanoic acid
C18:0	Stearic acid
C18:1n9	Oleic acid
C18:2n6	Linoleic acid
C18:3n3	Linolenic acid
C20:0	Arachidic acid
C20:1	*cis*-11-Eicosenoic acid
C20:2	*cis*-11,14-Eicosadienoic acid
C20:3n3+C21:0	*cis*-11,14,17-Eicosatrienoic acid and Heneicosanoic acid
C20:5n3	*cis*-5,8,11,14,17-Eicosapentaenoic acid
C22:0	Behenic acid
C22:1n9	Erucic acid
C23:0	Tricosanoic acid
C24:0	Lignoceric acid
C24:1	Nervonic acid

Table S2. Relevant protein and enzyme target coordinates of the docking box.

Group	Target	PDB ID	Grid size X, Y, Z	X, Y, Z dimensions	Reference
L. monocyto-genes	LPI-PLC	1AOD	64 Å X 94 Å X 108 Å	30.236, 37.512, 21.541	(Deepasree et al., 2023) [52]
	InlA	1O6T	88 Å X 70 Å X 50 Å	-5.921, -11.917, 4.481; -7.839,15.509, 64.698	(Venugopal, 2024) [53]
	PrfA	6EXL	40 Å x 40 Å x 40 Å	-17.276, -14.527, 9.533	center of inhibitor
B. cereus	Bc II	1BVT	64 Å x 64 Å x 64 Å	8.11, 33.514, 46.508	(Zhang et al., 2017) [55]
	PatB1	5V8D	84 Å x 96 Å x 118 Å	237.171, -4.531, 113.48	(Yu et al., 2010) [54]
	PadR	4ESF	84 Å x 62 Å x 56 Å	3.181, 18.166, -4.846	(Yu et al., 2010) [54]
	TubR	6AHT	68 Å x 80 Å x 50 Å	6.438, 55.563, 12.173	(Yu et al., 2010) [54]
P. aeruginosa	FabZ	1U1Z	84 Å x 66 Å x 58 Å	16.727,42.035, 134.039	(Yu et al., 2010) [54]
	LasR	2UV0	40 Å x 40 Å x 40 Å	23.346, 15.093, 80.969	(Baburam et al., 2022) [56]
	LpxC	3UHM	82 Å x 100 Å x 112 Å	1.05, 0.051, 2.522	(Zuo et al., 2017) [58]
	BETA LACTAMASE OXA-10	1FOF	80 Å x 80 Å x 126 Å	34.311, 39.934, 64.728	(Malathi et al., 2016) [57]
S. aureus	30S ribosome S3	5tcu	76 Å x 76 Å x 76 Å	99.46, 230.082, 201.387	(Saqallah et al., 2022) [60]
	Dihydropteroate syn-thase	1ad4	60 Å x 60 Å x 60 Å	32.46, 6.683, 42,972	(Saqallah et al., 2022 [60]; Hetmann et al., 2023 [56])
	Gyrase B	4urn	60 Å x 60 Å x 40 Å	-31.684, -5.252, 1.572	(Saqallah et al., 2022 [60]; Hetmann et al., 2023 [56])
	MurE	4c13	60 Å x 60 Å x 60 Å	-23.122, 2.508, 9.873	(Saqallah et al., 2022 [60]; Hetmann et al., 2023 [56])
	Transpeptidase	5tw8	60 Å x 60 Å x 60 Å	21.390, -62.210, 39.196	(Saqallah et al., 2022) [60]
E. coli	30S ribosome S3	4v53	88 Å x 88 Å x 88 Å	130.966, 32.099, 0.385	(Saqallah et al., 2022 [60]; Hetmann et al., 2023 [56])
	Dihydropteroate syn-thase	5v7a	60 Å x 60 Å x 60 Å	-17.836, -17.836, 103.740	(Saqallah et al., 2022) [60]
	Gyrase B	1kzn	60 Å x 60 Å x 60 Å	12.467, 27.336, 44.916	(Saqallah et al., 2022 [60]; Hetmann et al., 2023 [56])
	MurE	1e8c	70 Å x 60 Å x 70 Å	45.098, 37.112, 76.674	(Saqallah et al., 2022 [60]; Hetmann et al., 2023 [56])
	Transpeptidase	6ntw	60 Å x 60 Å x 60 Å	16.929, -32.370, 42.151	(Saqallah et al., 2022 [60]; Hetmann et al., 2023 [56])
Colon Adenocarcinoma	c-FOS	1fos	62 Å X 76 Å X 64 Å	55.088, -4.748, -8.684	(Yu et al., 2010) [54]
	CDK4	2w96	42 Å X 64 Å X 74 Å	5.126, -5.071, 74.401	(Karthick et al., 2024) [72]
	CDKN1A	5e0u	58 Å X 48 Å X 64 Å	-4.204, 52.009, -8.555	Center of inhibitor
	CyclinD1	2w99	26 Å X 30 Å X 26 Å	15.991, 26.615, 49.003	(Yu et al., 2010) [54]
	IFN-γ	1fg9	70 Å X 60 Å X 78 Å	33.23, 1.815, 6.433	(Yu et al., 2010) [54]
	IL-2	2erj	40 Å X 46 Å X 44 Å	23.659, 54.714, -17.097	(Yu et al., 2010) [54]
	IRS-1	1irs	106 Å X 106 Å X 106 Å	-0.325, -0.542, -0.406	(Yu et al., 2010) [54]
	NF-κB p65	5u4k	30 Å X 22 Å X 24 Å	6.313, 13.606, -7.004	(Yu et al., 2010) [54]

	TGF-β1	5vqp	94 Å X 64 Å X 96 Å	80.405, 44.585, 34.318	(Sun et al., 2023) [61]
	E2F1	2aze	40 Å X 40 Å X 40 Å	52.322, 24.479, 36.289	(Yu et al., 2010) [54]
	AKT-1	4vg1	20 Å X 38 Å X 34 Å	-17.903, 0.789, 15.945	(Yu et al., 2010) [54]
Lung Adenocarcinoma	CDK2	6gue	40 Å X 40 Å X 40 Å	-6.463, -24.101, 25.309	Center of inhibitor
	cIAP1BIR3	4kmn	50 Å X 50 Å X 50 Å	0.277, 53.967, 13.906	Center of inhibitor
	EGFR	1m17	64 Å X 34 Å X 28 Å	25.126,1.027, 51.326	Center of inhibitor
	MDM2	4wt2	40 Å X 50 Å X 40 Å	8.697, -28.139, -5.25	Center of inhibitor
	PI3K delta	4xe0	40 Å X 40 Å X 40 Å	-5.632, -11.8, 21.568	Center of inhibitor
	Stat3	6njs			(Yu et al., 2010) [54]

Table S3. Relevant protein and enzyme result of the docking scores

Group	Compound	PDB ID	Binding energy	PDB ID	Binding energy	PDB ID	Binding energy
L. monocytogenes	Linolenic acid	1AOD	-4.6	1O6T	-6.1	6EXL	-7.2
	Palmitic acid	1AOD	-3.7	1O6T	-4.5	6EXL	-6.5
	Oleic acid	1AOD	-3.7	1O6T	-5	6EXL	-6.6
	Palmitoleic acid	1AOD	-3.3	1O6T	-4.8	6EXL	-6.6
	Linoleic acid	1AOD	-3.6	1O6T	-5.4	6EXL	-7.2
B. cereus	Linolenic acid	1BVT	-4.7	5V8D	-4.2	4ESF	-3.8
	Palmitic acid	1BVT	-4.2	5V8D	-3.1	4ESF	-3.7
	Oleic acid	1BVT	-4.8	5V8D	-3.8	4ESF	-4.2
	Palmitoleic acid	1BVT	-4.7	5V8D	-3.9	4ESF	-4.2
	Linoleic acid	1BVT	-4.9	5V8D	-3.8	4ESF	-4.5
	Linolenic acid	6AHT	-5.1				
	Palmitic acid	6AHT	-4.7				
	Oleic acid	6AHT	-4.4				
	Palmitoleic acid	6AHT	-4.3				
	Linoleic acid	6AHT	-4.2				
P. aeruginosa	Linolenic acid	1U1Z	-4.9	2UV0	-4.8	3UHM	-4.9
	Palmitic acid	1U1Z	-3.5	2UV0	-4.3	3UHM	-3.1
	Oleic acid	1U1Z	-4	2UV0	-7.7	3UHM	-4.5
	Palmitoleic acid	1U1Z	-4.2	2UV0	-4.4	3UHM	-4.1
	Linoleic acid	1U1Z	-4.3	2UV0	-4.5	3UHM	-3.4
	Linolenic acid	1FOF	-4.1				
	Palmitic acid	1FOF	-3.8				
	Oleic acid	1FOF	-4.4				
	Palmitoleic acid	1FOF	-3.6				
	Linoleic acid	1FOF	-3.6				
S. aureus	Linolenic acid	5tcu	-4.6	1ad4	-4.5	4urn	-4.3
	Palmitic acid	5tcu	-3.8	1ad4	-3.8	4urn	-4.3
	Oleic acid	5tcu	-4.4	1ad4	-3.6	4urn	-3.7
	Palmitoleic acid	5tcu	-3.9	1ad4	-4.2	4urn	-4.7

	Linoleic acid	5tcu	-4.2	1ad4	-3.7	4urn	-4.5
	Linolenic acid	4c13	-4.6	5tw8	-5.7		
	Palmitic acid	4c13	-4.8	5tw8	-4.5		
	Oleic acid	4c13	-5.4	5tw8	-4.3		
	Palmitoleic acid	4c13	-4.2	5tw8	-4.5		
	Linoleic acid	4c13	-5.1	5tw8	-5.2		
E. coli	Linolenic acid	4v53	-4.5	5v7a	-4.6	1kzn	-4.7
	Palmitic acid	4v53	-3.6	5v7a	-3.9	1kzn	-4.6
	Oleic acid	4v53	-3.9	5v7a	-3.9	1kzn	-4.2
	Palmitoleic acid	4v53	-4.5	5v7a	-3.9	1kzn	-5.1
	Linoleic acid	4v53	-4.1	5v7a	-3.6	1kzn	-4.7
	Linolenic acid	6ntw	-4.8	1e8c	-5.4		
	Palmitic acid	6ntw	-4.1	1e8c	-4.7		
	Oleic acid	6ntw	-4.7	1e8c	-4.5		
	Palmitoleic acid	6ntw	-4.2	1e8c	-4.3		
	Linoleic acid	6ntw	-4.8	1e8c	-4.5		
Colon Adenocarcinoma	Linolenic acid	1fos	-3.4	2w96	-4.1	5e0u	-5.2
	Palmitic acid	1fos	-3	2w96	-4.9	5e0u	-5
	Oleic acid	1fos	-3.1	2w96	-4.8	5e0u	-5.1
	Palmitoleic acid	1fos	-3.2	2w96	-5.3	5e0u	-4.5
	Linoleic acid	1fos	-2.9	2w96	-4.7	5e0u	-4.5
	Linolenic acid	2w99	-5.3	1fg9	-5.2	2erj	-5
	Palmitic acid	2w99	-4.8	1fg9	-4	2erj	-4.4
	Oleic acid	2w99	-5	1fg9	-4.6	2erj	-4.2
	Palmitoleic acid	2w99	-4.9	1fg9	-4.7	2erj	-4
	Linoleic acid	2w99	-5.2	1fg9	-4.9	2erj	-3.5
	Linolenic acid	1irs	-3.4	5u4k	-3.6	5vqp	-4
	Palmitic acid	1irs	-2.7	5u4k	-2.6	5vqp	-3.7
	Oleic acid	1irs	-3	5u4k	-3.2	5vqp	-3.5
	Palmitoleic acid	1irs	-3.2	5u4k	-2.8	5vqp	-3.3
	Linoleic acid	1irs	-3	5u4k	-3	5vqp	-3.9
	Linolenic acid	2aze	-3.6				

	Palmitic acid	2aze	-3				
	Oleic acid	2aze	-3.3				
	Palmitoleic acid	2aze	-3.4				
	Linoleic acid	2aze	-3.6				
Lung Adenocarcinoma	Linolenic acid	4vg1	-5.3	6gue	-5.9	4kmn	-3.8
	Palmitic acid	4vg1	-4.7	6gue	-5.7	4kmn	-4.2
	Oleic acid	4vg1	-4.9	6gue	-6.1	4kmn	-3.8
	Palmitoleic acid	4vg1	-4.9	6gue	-5.9	4kmn	-3.9
	Linoleic acid	4vg1	-6	6gue	-6.3	4kmn	-3.6
	Linolenic acid	1m17	-5.3	4wt2	-6.4	4xe0	-6.5
	Palmitic acid	1m17	-4.6	4wt2	-5.4	4xe0	-5.5
	Oleic acid	1m17	-5.3	4wt2	-6	4xe0	-5.8
	Palmitoleic acid	1m17	-5.2	4wt2	-6.1	4xe0	-5.4
	Linoleic acid	1m17	-5.3	4wt2	-6.6	4xe0	-6.2
	Linolenic acid	6njs	-4.9				
	Palmitic acid	6njs	-4.2				
	Oleic acid	6njs	-4.8				
	Palmitoleic acid	6njs	-4.7				
	Linoleic acid	6njs	-4.9				

Table S4. Selected protein-ligand complexes for MM/PBSA binding free energy analysis based on molecular dynamics simulations.

Complex	Frames	VDWAALS	EEL	EGB	ESURF	GGAS	GSOLV	TOTAL
Palmitic acid_PrfA	Average	-35.08	-9.59	25.34	-5.86	-44.67	19.48	-25.19
	SD	3.93	5.84	4.26	0.56	7.11	3.93	4.09
	SEM	0.13	0.19	0.14	0.02	0.24	0.13	0.14
Oleic acid_LasR	Average	-36.24	64.24	-43.92	-6.44	28	-50.36	-22.36
	SD	4.56	21.09	18.44	0.73	20.91	18.17	4.81
	SEM	0.15	0.7	0.61	0.02	0.7	0.61	0.16
Palmitoleic acid_EGFR	Average	-34.94	33.52	-10.48	-5.68	-1.43	-16.16	-17.58
	SD	4.27	44.54	38.31	0.59	43.43	38.23	7.09
	SEM	0.14	1.48	1.28	0.02	1.45	1.27	0.24
Linolenic acid_AKT-1	Average	-23.54	-36.07	45.75	-4.86	-59.6	40.89	-18.71
	SD	3.01	16.33	15.38	0.33	15.03	15.48	3.56
	SEM	0.91	4.92	4.64	0.1	4.53	4.67	1.07
